# Cauliflower Excrescence—Portion of Peritoneum Removed

**Published:** 1875-04

**Authors:** 


Cauliflower Excrescence—Portion of Peritoneum Removed—Prof. Karl Braun says: Experience has conclusively proven that nothing short of its radical removal will be of any value. Any attempt short of this will result in a failure, and the condition of the patient subsequently will be even worse than at present. Here it is always better to cut away too much tissue than not enough. A portion of the peritoneum may be removed, proper care being taken in the subsequent treatment, and yet no injury result. In the very many cases here operated upon in the past twenty years, but a very small percentage has been lost, while in many of these the peritoneum has been cut. I operate every year fifteen or twenty times for this difficulty, and often remove a portion of the peritoneum so large that my assistants scratch their heads and say, "He has gone too far this time;" but as they grow older and see these operations again and again repeated with most favorable results, they learn better. The surgeon would object to such an operation, involving the opening of the peritoneal sac, but the gynæcologist, who operates in this region much oftener, knows better what can be done. * * *

You will observe, gentlemen, as this specimen passes around the class, that I have removed quite a considerable portion of peritoneum. It is better to avoid doing this, if possible. I do not think this case required it, and had not intended to touch it, but the folds of the posterior wall of the vagina deceived me. Our subsequent treatment will be very simple. We will use a glycerin tampon, taking care not to introduce it so far that there would be danger of its passing into the abdominal cavity; and and no injection whatever will be allowed. The patient will be required to lie as much as possible on her back, the severed tissues being thus thrown more nearly into accurate juxtaposition, and the healing process will be proportionately more rapid.

Nov. 14.—Patient doing very well. There has been no rise in temperature since the operation.—*Medical Times.*

